# Associated factors with mammographic changes in women undergoing breast cancer screening

**DOI:** 10.1590/S1679-45082016AO3708

**Published:** 2016

**Authors:** Ricardo Soares de Sant'Ana, Jacó Saraiva de Castro Mattos, Anderson Soares da Silva, Luanes Marques de Mello, Altacílio Aparecido Nunes

**Affiliations:** 1 Hospital de Câncer de Barretos, Barretos, SP, Brazil; 2 Faculdade de Medicina de Ribeirão Preto, Universidade de São Paulo, Ribeirão Preto, SP, Brazil

**Keywords:** Breast neoplasms, Mammography, Obesity, Mass screening

## Abstract

**Objective:**

To evaluate association of sociodemographic, anthropometric, and epidemiological factors with result of mammogram in women undergoing breast cancer screening.

**Methods:**

This is a cross-sectional study with data obtained through interviews, anthropometric measurements, and mammography of 600 women aged 40 to 69 years at the Preventive Medicine Department of *Hospital de Câncer de Barretos*, Brazil, in 2014. The results of these examinations in the BI-RADS categories 1 and 2 were grouped and classified in this study as normal mammogram outcome, and those of BI-RADS categories 3, 4A, 4B, 4C, and 5 were grouped and classified as altered mammogram outcome. The statistical analysis included the Student's *t*-test to compare means, as well as odds ratios (OR), with their corresponding 95% confidence intervals (95%CI), to verify an association by means of the multivariate analysis.

**Results:**

Of 600 women evaluated, 45% belonged to the age group of 40–49 years-old and 60.2% were classified as BI-RADS category 2. The multivariate analysis showed that women with blood hypertension (OR: 2.64; 95%CI: 1.07–6.49; p<0.05) were more likely to present changes in the mammography, while physical activity was associated with lower chances (OR: 0.30; 95%CI: 0.11–0.81; p<0.05).

**Conclusion:**

Hypertensive women undergoing screening mammography are more likely to present mammographic changes, whereas women practicing physical activity have lower chances (70%) of presenting changes in the breast compared with sedentary individuals.

## INTRODUCTION

Breast cancer is a multifactorial disease and its several risk factors related to the disease are well known, and they become worse with changes in life habits. Risk factors include age, genetics, family history, endocrine and reproductive factors, alcohol consumption, fat-rich diet, obesity, sedentarism, exposition to ionizing radiation, pre-malignant injuries of the breast and high density of breast tissue. The factors associated with women's reproductive life and longer exposition to endogen estrogens, such as early menarche, nulliparity, use of hormone replacement therapy for more than five years and late age at first delivery are among most important risk factors for breast cancer.^(1,2)^

Some factors are inevitable, such as age and family history, however, in almost 30% of cases of breast cancer factors can be avoided by adoption of some habits, *e.g.* healthy eating, regular exercise, adequate weight, moderate alcohol consumption, and non-smoking.^(1)^ Age remains one of the most important risk factors, and incidence growth rapidly up to 50 years and, after this age range, the increase is more moderately.^(2)^

Mammography is currently considered one of the most reliable techniques to early detection of breast cancer, comprising an ideal method to identify subclinical injuries. Among actions that favor early detection of disease, there are clinical breast exams and screening mammography in routine of integral health care for women. According to the outcome, the exam can be classified in screening mammography that is directed to asymptomatic women, and diagnostic mammography recommended for those with signs and symptoms of breast cancer.^(1,2)^ Although not incorporated in routine of public health service, the annual mammography is ensured as right in Brazilian Public Health Services (SUS - *Sistema Único de Saúde*) to all women aged 40 years or older according to the Federal Law nº 11,664, April 29, 2008, that come into effect in 2009.^(3)^ A study suggests annual mammography before diagnosis of breast cancer as a predictor of higher global survival,^(4)^ and, therefore, the screening results in real benefits to the patient with decrease in mortality for this type of cancer.^(5,6)^

Regular exercise is associated with better prognosis and survival in women with breast cancer, in addition it has a protective role against several types of cancer, because it prevents obesity, which is one of the risk factors for the disease. Studies on exercises and their biologic mechanisms shown them as protective factor for breast cancer and this result have increased considerably in the last years.^(7–9)^ The American College of Sports Medicine recommends for general population, a total of 150 minutes weekly of cardiorespiratory exercises in order to reduce mortality and enhance quality of life.^(10)^ However, recommendations regarding level of physical activity (LPA) to reduce breast cancer risk are little longer, the suggestion is 180 minutes of exercises with moderate intensity weekly to reduce risk approximately in 3%.^(11)^

## OBJECTIVE

To evaluate association of sociodemographic, anthropometric and epidemiologic factors with results of mammograms of women who underwent breast cancer screening.

## METHODS

This cross-sectional study obtained data from interviews, anthropometric assessments and results of mammograms of women assisted at Department of Prevention of *Hospital de Câncer de Barretos* (HCB) from July 1, 2014 to November 3, 2014.

The study was approved by the Ethical and Research Committee of the *Hospital das Clínicas da Faculdade de Medicina de Ribeirão Preto* at *Universidade de São Paulo* and Ethical Committee of HCB, under protocol number 1.169.686 CAAE: 24514413.1.0000.5440. Participants signed the Consent Form, authorized data collection, and publication of results.

In the HCB women aged 40 years or older underwent screening mammograms. In this sense, considering simple random sample with limited population, based in number of exams conducted in the mentioned service (18,000 per year) and prevalence of obesity in Brazil in adult women of 16.9%, estimating a tolerable absolute error of 3% and significance level of 5%, our total sample was composed by 583 women. However, we evaluated 600 women aged between 40 and 69 years who were previously identified to perform screening mammography.

We excluded pregnant women who had pacemakers and who had positive diagnosis for any other type of cancer and presented some type of physical impairment and cognitive *deficit*.

Interview was carried out in the same day of the mammography using a structured questionnaire. The assessment of level of physical activity practice was applied based in International Physical Activity Questionnaire that measures level of activity and classifies individuals as inactive, irregular active, active and very active. We classified anthropometrical assessment including height measure, body weight, body mass index (BMI) and waist and hip circumference. After finishing data collection, anthropometric assessments and mammography exam, we initiated the follow-up and analysis of report of the exam in which results of breast density and mammography classification were obtained. Result of this exam was interpreted by radiologists according to standardized system known as Breast Imaging Reporting and Data System Mammography (BI-RADS^®^) of the American College of Radiology.^(12)^

In our study, categories of BI-RADS were classified as incomplete mammography assessment (BI-RADS 0), in which there was need of additional assessment of image or previous mammograms for comparison and complete assessment (final categories), involving BI-RADS 1–5.

Results of mammograms in categories BI-RADS 1 and 2 were grouped and classified as “normal mammography finding”. These referred to exams without mammographic findings or those with benign (however with some change). But results of categories BI-RADS 3, 4A, 4B, 4C and 5 were grouped and classified as “altered mammogram finding”, in which entailed benign results (however with need of follow-up exams), findings with low suspicion of malignity, intermediate suspicion of malignancy, moderate and high suspicion of malignancy.

Classification of breast density was based in proportion of tissues that composed breast that was described by Wolfe^(13)^ using four patterns: N1 – primarily fat, all breast composed almost with fat; P1 – fibroglandular tissue pattern with prominent ducts occupying up to one-third of the breast volume; P2 -fibroglandular tissue pattern with prominent ducts occupying more than one-fourth of breast volume, DY; fibroglandular pattern occupying almost all the breast. Patterns N1 and P1, characterized in this study as fat breast and primarily fat breast were considered low density, whereas P2 and DY were considered as dense and predominantly dense with high risk for development of breast cancer.

### Data analysis

Responses related to sociodemographic, epidemiologic, clinical and anthropometric parameters were analyzed in relation to association among independent variables such as BMI and breast density, and dependents (BI-RADS categories, normal mammographic findings and altered mammogram finding). To measure and compare degree of linear correlation between variables of BMI and waist-hip circumference ratio, we applied coefficient of Pearson correlation. The χ^2^ test was used to analyze data concerning differences among categorical variables.

Comparisons between normal distribution parameters were analyzed by Student's *t* test for independent or dependent samples, when indicated. Parameters without normal distribution or with unknown distribution were compared using the Mann-Whitney test. To evaluate association among obesity, excessive weight, socioeconomic, epidemiologic, anthropometric variables and health we employed univariated and multivariated analyses, considering altered mammogram finding as dependent variable and applying prevalence ratio and odds ratio (OR) with respective confidence interval of 95% (CI95%). The level of significant adopted in all analyses was 5%.

## RESULTS

The age of the 600 women included in the study ranged from 40 to 69 years, mean of 51.7 years (±7.99). Participants' mean weight was 72.2 (±14.40) kg and mean height was 1.57 (±0.06) m. Concerning obesity classified by BMI, the mean was 29.38 (±5.62) kg/m^2^, whereas waist circumference was 93.0 (±12.57) cm and hip circumference was 103.7 (±9.84) cm. The mean of waist-hip circumference ratio was 0.89 (±0.08) cm. Most of participants were aged between 40 to 49 years (45.0%), followed by 50 to 59 years (34.8%). The majority of women selected were irregularly activity (47.5%) whereas those active or very active represented a total of 34.7% and 3.6%, respectively. We also verified a predominance of women with primarily dense breasts (60.7%).

In relation to BMI (Table 1), a higher percentage of obese women was seen (40.5%), and only 21.5% were within the BMI considered normal. We also observed high percentage of overweight women (78.9%), but few participants (14.7%) were classified as normal for abdominal obesity and predominant significant enlarged risk were seen in 64.8% of participants. Concerning, there waist-hip circumference ratio was predominance of high risk and very high risk of 87.7% in the sample.

**Table 1:** Distribution of anthropometrical characteristics of participants according to body mass index

Variables		n (%)
Classification by BMI		
	Underweight	3 (0.5)
	Normal	129 (21.5)
	Overweight	225 (37.5)
	Obesity degree I	147 (24.5)
	Obesity degree II	72 (12.0)
	Obesity degree III	24 (4.0)
Excess weight		
	Overweight	225 (37.5)
	Obesity	243 (40.5)
Abdominal obesity		
	Normal	88 (14.7)
	Enlarged risk	123 (20.5)
	Substantial risk	389 (64.8)
Waist-hip circumference ratio		
	Low risk	6 (1.0)
	Moderate risk	68 (11.3)
	High risk	195 (32.5)
	Very high risk	331 (55.2)
Total		600 (100.0)

BMI: body mass index.

In table 2 we can observe data concerning results of mammograms according to BI-RADS^®^ classification. We also observed that only 7.4% of exams were classified in BI-RADS 0 category, suggesting an additional evaluation with other complementary exam. Among women who underwent mammography in the period of investigation, 94.8% had their exams classified as normal in BI-RADS 1 and 2 classification, after reclassification of BI-RADS 0. Other categories of BI-RADS entailed 5.2% of the sample.

**Table 2:** Results of mammograms based on BI-RADS classification

BI-RADS categories	Right breast	Left breast	Total	Changes in both breasts
	n (%)	n (%)	n (%)	n (%)
0	33 (5.5)	56 (9.3)	89 (7.4)	0 (0.0)
N1	206 (34.3)	209 (34.8)	415 (34.6)	0 (0.0)
2	362 (60.3)	361 (60.2)	723 (60.2)	0 (0.0)
3	23 (3.8)	17 (2.8)	40 (3.3)	40 (64.5)
4A	4 (0.7)	9 (1.5)	13 (1.1)	13 (21.0)
4B	1 (0.2)	4 (0.7)	5 (0.4)	5 (8.1)
4C	3 (0.5)	0 (0.0)	3 (0.3)	3 (4.8)
5	1 (0.2)	0 (0.0)	1 (0.1)	1 (1.0)
Total	633 (100.0)	656 (100.0)	1289	62 (100.0)

Comparison of women's mean age among those with normal mammographic findings and altered mammogram finding of right breast (Table 3), did not show difference between groups (p<0.05). However, the comparison of mean women's age with normal mammographic findings and altered mammogram finding of left breast that showed difference between the two groups (p<0.05). In comparison of mean value of BMI in women with normal mammographic findings and altered mammogram finding of right and left breast no significant difference was seen (p>0.05).

**Table 3:** Comparison of mean age and body mass index in women from the study considering normal and abnormal mammogram findings

Mammographic finding	Mean age in years (±SD)	p value
Normal right breast	51.76 (±8.04)	>0.05
Altered right breast	51.75 (±7.17)	
Normal left breast	51.92 (±7.97)	<0.05*
Altered left breast	48.70 (±7.70)	
	Mean BMI (±SD)	p value
Normal right breast	29.38 (±5.55)	>0.05
Altered right breast	29.40 (±6.81)	
Normal left breast	29.47 (±5.71)	>0.05
Altered left breast	27.69 (±3.10)	

* Student's *t* test for independent samples.

SD: standard deviation; BMI: body mass index.

In table 4, we observed that presence of systemic arterial hypertension (SAH) appeared as a predictor of changing in mammography because in multivariate analysis this finding suggested that hypertensive women have 2.64 times more chance (OR: 2.64; 95%CI: 1.07–6.49; p<0.05) of presenting changes in the breast compared with non-hypertensive individuals. However, the LPA can indicate that active women had 70% (OR: 0.30; 95%CI: 011–0.81) less changes in mammograms compared with little active or inactive women.

**Table 4:** Multivariate analysis of factors associated with mammograms (normal and abnormal) among selected women, left and right breast

Variables	OR	95%CI
RBD	0.09	0.01–1.05
LBD	19.74	1.82–23.45
Marital status	0.54	0.19–1.47
Race	2.18	0.92–5.16
Age range	0.65	0.28–1.51
SAH	2.64	1.07–6.49
BM	0.85	0.23–3.10
LPA	0.30	0.11–0.81
Abdominal obesity	0.40	0.08–1.91
Waist-hip circumference ratio	12.73	0.76–214.08

OR: odds ratio; 95%CI: 95% confidence interval; RBD: right breast density; LBD: left breast density; SAH: systemic arterial hypertension; BMI: body mass index; LPA: level of physical activity.

## DISCUSSION

Breast cancer is one of the neoplasia that more affects women in Brazil, excluding non-melanoma skin cancer, and it is one of the main causes of death from cancer in developed and under developing countries. Although enhancements in breast cancer screening service had occurred, and advances in development of early diagnosis methods and treatment in developed countries, this disease represents the main cause of death among women affected by cancer in many countries, mainly in less developed ones.^(14,15)^

Population screening program are important and efficient to reduce breast cancer mortality because it might detect potential diseases in early stages.^(15)^ Screening with mammography test is adopted as a public health strategy, and it is considered the best method for early diagnosis of breast cancer.^(16)^ Studies suggest that annual mammography before diagnosis of breast cancer is a predictor of increase in global survival, however, these studies highligth that methods and programs to early detection of breast cancer do not reduce the disease incidence, but they can reduce the disease mortality.^(17,18)^

The Haikel et al.^(19)^ study about a screening based program in mammography reported strategies on adherence and better optimization in breast cancer detection using this initiative. For these authors, administrators of public health institutions must be always encouraged to adhere to population level program, which might consequently increase participation of these women.

The analysis of obesity prevalence in our sample revealed that most of women were classified as obese (40.5%), and 37.5% as overweight, *i.e.*, excessive weight was seen in 78.0% of the sample. A study including 145 women without breast cancer who participated in a screening program in South Brazil found a prevalence of obesity of 39.3% and excessive weight of 76.5%. The study emphasized these alarming rates and highlighted as a possible explanation for high prevalence of excessive weight other associated factors such as low education level and no exercise.^(20)^ Of note is that some studies report categorical data that excessive body fat represents a risk factor to increase incidence of breast cancer, particularly among women in postmenopausal period.^(21,22)^

Based on this fact, it is necessary to identify how obesity can affect and change organism of these women. Obesity is correlated with a number of changes in the organism and, therefore, can affect prognosis of breast cancer.^(23)^ One of this changes is the increase of circulating estrogen levels: an aromatase enzyme found in adipocytes and responsible for conversion of androstenedione into estrogen.^(24)^ Other studies have demonstrated that most of mediating effect of estrogen represent the association of BMI and breast cancer in such a way that estrogen stimulate the division of epithelial breast cells, therefore increasing the risk for mutation, and for this reason, favoring and inducing breast cancer. In addition, hyperinsulinemia which is closely related with obesity, reduces level of sexual hormones binding globulin, and cause an increase in levels of estradiol and testosterone.^(25)^

In our study, the sample was characterized by LPA such as inactive, irregularly active, active and very active participants. We observed a predominance of women categorized and classified as sedentary (61.7%, inactive and irregularly active participants). By multivariate analysis, we observed an association of LPA with mammography finding, active women present 70% lower chance of presenting changes in breast compared with sedentary patients. Based on this fact, physical activity seems to be associated with a protective factor against changes in the breast. According to some international agencies such as World Cancer Research Fund and American Institute of Cancer Research, the regular practice of exercise at least 30 minutes a day is considered a protective factor for breast cancer in postmenopausal period, in addition to help to maintain the healthy level of some hormones. However, evidences of protective effect of this variable are still limited for women in pre-menopause period.^(26)^ Although results did not show a relationship among obesity with changes and prevalence of changes in the breast, the lifestyle, exercise, and eating habits are considered important factors to prevent and reduce breast cancer risks.

Among findings we highlight the SAH in which found an association with change in breast, suggesting that women with hypertension have 2.6 times more chance of presenting changes in the breast compared with normotensive women. A recent study including women with breast cancer found similar prevalence of comorbidities, and it was higher in SAH with 32.8%, thyroid 22.4% and hypercoloesterolemia with 12.7%.^(27)^ Another large study on metabolic syndrome and initial stages of breast cancer identified higher risk of general mortality attributed to SAH, however, it was not possible to find the same association for breast cancer.^(28)^

Contribution of several modifiable risk factors for global load of breast cancer, except the reproductive, was calculated by some authors, and they conclude that 21% of all deaths by breast cancer around the world are attributed to alcohol consumption, overweight, obesity and physical inactivity. This proportion was higher in countries with high income (27%), and the most important influencers are overweight and obesity. In countries with mean and low income the proportion of breast cancer attributed to these findings was 18%, and physical inactivity was the most important determinant (10%).^(29)^ However, policies and national programs must be implemented to increase conscience and reduce exposition to risk factors of cancer, and also guarantee provision of information for individuals and support they would need to adopt healthy life habits.^(30)^

## CONCLUSION

Systemic arterial hypertension variables and level of physical activity had significant association with changed mammographic finding. Results showed that although prevalence of overweight and obesity among women in the sample was above the expected for this population, the body mass index did not show significant difference in association with mammographic findings. In relation to level of physical activity, this finding suggested that women considered active had 70% lower chance of presenting change in the breast compared with sedentary women.
